# Design of novel multiepitope constructs-based peptide vaccine against the structural S, N and M proteins of human COVID-19 using immunoinformatics analysis

**DOI:** 10.1371/journal.pone.0240577

**Published:** 2020-10-15

**Authors:** Niloofar Khairkhah, Mohammad Reza Aghasadeghi, Ali Namvar, Azam Bolhassani

**Affiliations:** 1 Department of Hepatitis, AIDS and Blood-borne diseases, Pasteur Institute of Iran, Tehran, Iran; 2 Iranian Comprehensive Hemophilia Care Center, Tehran, Iran; University of Massachusetts Medical School, UNITED STATES

## Abstract

The causative agent of severe acute respiratory syndrome (SARS) reported by the Chinese Center for Disease Control (China CDC) has been identified as a novel *Betacoronavirus* (SARS-CoV-2). A computational approach was adopted to identify multiepitope vaccine candidates against SARS-CoV-2 based on S, N and M proteins being able to elicit both humoral and cellular immune responses. In this study, the sequence of the virus was obtained from NCBI database and analyzed with *in silico* tools such as NetMHCpan, IEDB, BepiPred, NetCTL, Tap transport/proteasomal cleavage, Pa^3^P, GalexyPepDock, I-TASSER, Ellipro and ClusPro. To identify the most immunodominant regions, after analysis of population coverage and epitope conservancy, we proposed three different constructs based on linear B-cell, CTL and HTL epitopes. The 3D structure of constructs was assessed to find discontinuous B-cell epitopes. Among CTL predicted epitopes, S^257-265^, S^603-611^ and S^360-368^, and among HTL predicted epitopes, N^167-181^, S^313-330^ and S^1110-1126^ had better MHC binding rank. We found one putative CTL epitope, S^360-368^ related to receptor-binding domain (RBD) region for S protein. The predicted epitopes were non-allergen and showed a high quality of proteasomal cleavage and Tap transport efficiency and 100% conservancy within four different clades of SARS-CoV-2. For CTL and HTL epitopes, the highest population coverage of the world’s population was calculated for S^27-37^ with 86.27% and for S^196-231^, S^303-323^, S^313-330^, S^1009-1030^ and N^328-349^ with 90.33%, respectively. We identified overall 10 discontinuous B-cell epitopes for three multiepitope constructs. All three constructs showed strong interactions with TLRs 2, 3 and 4 supporting the hypothesis of SARS-CoV-2 susceptibility to TLRs 2, 3 and 4 like other Coronaviridae families. These data demonstrated that the novel designed multiepitope constructs can contribute to develop SARS-CoV-2 peptide vaccine candidates. The *in vivo* studies are underway using several vaccination strategies.

## Introduction

The causative agent of severe acute respiratory syndrome (SARS) reported by the Chinese Center for Disease Control (China CDC) has been identified as a novel *Betacoronavirus* (SARS-CoV-2) [1]. The genomic sequence of SARS-CoV-2 was similar but its composition was diverse as compared to SARS-CoV’s and MERS-CoV’s genome [2]. Accumulated clinical and experimental knowledge on these previous coronaviruses has led to an easier prediction of host immune responses against this particular virus. Genomic RNA of SARS-CoV-2 encodes non-structural replicase polyprotein and structural proteins including spike (S), envelope (E), membrane (M) and nucleocapsid (N). The entry of SARS-CoV-2 into host cells is mediated by attachment of S glycoprotein on the virion surface to the angiotensin-converting enzyme 2 (ACE2) receptor [3] mainly expressed in type 2 alveolar cells of lungs [4]. Enhanced binding affinity between SARS-CoV-2 and ACE2 receptor was proposed to correlate with increased virus transmissibility [5]. The trimeric S protein will be cleaved into two subunits of S1 and S2 during viral infection [6]. S1 and S2 subunits are responsible for binding to the ACE2 receptor and the fusion of the viral and cellular membranes, respectively [3]. Being the main antigenic component, S protein has been selected as an important target for vaccine development.

Anti-viral drugs, broad-spectrum antibiotics such as Remdesivir, Chloroquine, Ribavirin, Favipiravir or Baricitinib are potential therapeutic strategies used to reduce the viral load [7] by blocking the SARS-CoV-2 replication [8, 9]. Recently, the plasma exchange using convalescent sera of COVID-19 showed promising results [10, 11]. Also, the monoclonal antibody (CR3022) binding with the spike receptor-binding domain of SARS-CoV-2 had the potential to be developed as a therapeutic candidate [12]. Efforts toward developing an effective vaccine have been ignited in many countries. Actually, several projects have been reported by companies and researchers to start SARS-CoV-2 vaccine development. There are different kinds of novel vaccines including DNA-based, viral vector-based, recombinant S protein-based, adenovirus-based, mRNA-based and peptide-based vaccines. The mRNA-1273 candidate, an encapsulated mRNA vaccine encoding S protein developed by Moderna (NCT04283461), the Ad5-nCov candidate, an adenovirus type 5 vector expressing S protein developed by CanSino Biologicals (NTC04313127), the INO-4800 candidate, a DNA plasmid encoding S protein developed by Inovio Pharmaceuticals (NCT04336410), the LV-SMENP-DC candidate, dendritic cells modified with lentiviral vector (NCT04276896), and the pathogen-specific aAPC candidate, an aAPC modified with a lentiviral vector (NCT04299724) both developed by Shenzhen Geno-immune Medical Institute are few vaccines in phase I of the clinical trial against SARS-CoV-2 [13].

However, each type of vaccine has a number of advantages and disadvantages. Although platforms based on DNA or mRNA are flexible and effective for antigen manipulation, peptide-based vaccines are customizable multipurpose therapeutics which does not have the implication of stability or translation [14] and by the use of multiepitope approach, a single peptide-based vaccine can be designed to target different strains [15]. Despite safety and cost-effectiveness, peptide-based vaccines are difficult to design. The epitope-mapping is a crucial but time-consuming step in the design of a peptide-based vaccine. That is why no peptide-based vaccine for SARS-CoV-2 has reached phase I clinical trial to date. A successful peptide-based vaccine comprises immunodominant B-cell and T-cell being able to induce strong and long-lasting immunity against the desired pathogen [16]. Thus, the understanding of epitope interaction with major histocompatibility complex (MHC) is necessary. In the current study, a computational approach was adopted to identify multiepitope vaccine candidates against SARS-CoV-2 based on S, N and M proteins.

## Materials and methods

### Collection of targeted proteins sequences

The reference sequences of the targeted proteins including S, N and M proteins of SARS-CoV-2 were obtained from the NCBI database and used as an input for more bioinformatics analyses.

### Linear B-cell epitope prediction

A successful vaccine must elicit strong cellular and humoral immune responses. Thus, it is important to show that the constructed immunogens are able to induce protective immunity. It should be considered that optimal peptide-based vaccines must be presented in a desired secondary structure of peptides in order to induce a specific humoral response. In this subsection, we used BepiPred-2.0 prediction module (http://www.cbs.dtu.dk/services/BepiPred-2.0/) for linear B-cell prediction of the conserved regions in S, N and M proteins of SARS-CoV-2 to produce the B-cell mediated immunity. In this study, epitope threshold value was set as 0.5 (the sensitivity and specificity of this method are 0.58 and 0.57, respectively) [17].

### T-cell epitope identification

The initial step on applying bioinformatics to design synthetic peptide vaccines is to determine whether epitopes are potentially immunoprotective. T-cell epitopes presented by MHC are linear form containing 12 to 20 amino acids. This fact facilitates modeling for the interaction of ligands and T-cells with accuracy [18]. Binding of the MHC molecule is the most selective step in the presentation of antigenic peptide to T-cell receptor (TCR).

For MHC class I, we adapted Artificial Neural Networks (NetMHCpan4.1 server (http://www.cbs.dtu.dk/services/NetMHCpan/) to predict high-potential T-cell epitopes. This server is meant to predict MHC I binding with accuracy of 90–95% [19, 20]. Human alleles were used and the threshold for NetMHCpan was set at 0.5% for strong binders and 2% for weak binders.

For MHC class II, we used NetMHCIIpan 4.0 server (http://www.cbs.dtu.dk/services/NetMHCIIpan/) [21] to predict potential interaction of helper T-cell epitope peptides and MHC class II. Human alleles were used and the threshold for strong and weak binders was set at 2% and 10%, respectively.

### Prediction of MHC class I peptide presentation pathway

Best ranked peptides extracted from NetMHCpan database were used in transporter associated with antigen presentation (TAP) transport efficacy and proteasomal cleavage analysis. In MHC class I presentation pathway, this section is as essential as binding affinity prediction. We employed NetCTL 1.2 server combined with Tap transport/proteasomal cleavage tools (http://www.cbs.dtu.dk/services/NetCTL) to assess the prediction of antigen processing through the MHC-I antigen presentation pathway. In this method, weight on C-terminal cleavage set on 0.15, and tap transport efficacy and epitope identification were set on 0.05 and 0.75, respectively.

### Conservancy analysis

Up to now, more than 16667 full-sequences of SARS-CoV-2 have been registered globally in GISAID database classified into four clades of V, G, S and O. To calculate the degree of conservancy of each epitope, IEDB epitope conservancy tool (http://tools.immuneepitope.org/tools/conservancy/) was employed [22]. This tool computes the degree of conservancy of an epitope within a given protein sequence set at a given identity level. In this study, we determined epitope conservancy of each protein including S, N and M obtained from GISAID database.

### Population coverage

Due to a phenomenon known as denominated MHC restriction of T-cell responses, selecting multiple epitopes with different HLA binding specificities will afford more increases in population coverage. Prediction based on HLA binding at population level in defined geographical regions where the peptide-based vaccine might be employed is essential. Since MHC polymorphisms are dramatically at different frequencies in different ethnicities, without careful consideration, a vaccine with ethnically biased population coverage will result. In this study, we used IEDB population coverage tool [23] (http://tools.iedb.org/population/) to assess the coverage rate of population for each epitope.

### Antibody-specific epitopes prediction

IgPred module [24] (https://webs.iiitd.edu.in/raghava/igpred/index.html) was developed for predicting different types of B-cell epitopes inducing different classes of antibodies. We used this server to identify epitope tendency for inducing IgG and IgA antibodies.

### Prediction of cytokine inducer peptides

It is important to understand that all MHC class II binders will not induce the same type of cytokines. Thus, we used IL-10 Pred [25] (http://crdd.osdd.net/raghava/IL-10pred/) and IFNepitope [26] webserver (http://crdd.osdd.net/raghava/ifnepitope/index.php) to predict Il-10 and Interferon-gamma inducing peptides, respectively. We used Support Vector Machine (SVM)-based model as prediction model in both servers. Other features including SVM threshold left at the default value. Through using these servers, we improved insight into the future *in vivo* studies.

### Allergenicity and cross-reactivity assessment

The prediction of potential allergenicity is an important step in safety assessment as proteins and polypeptides have significant roles in inducing allergenic reactions. The allergenicity of the selected epitopes was calculated by PA^3^P server (http://lpa.saogabriel.unipampa.edu.br:8080/pa3p/pa3p/pa3p.jsp) using AFDS-motif, Allergen online (6aa and 80-word match) algorithms. The specificity of these methods is 95.43% (6aa), 92.88% (80aa), and 88.1% (ADFS) [27].

### Peptide-protein flexible docking

To estimate the formation of MHC-peptide complex, we used GalexyPepDock peptide-protein flexible docking server [28] (http://galaxy.seoklab.org/cgi-bin/submit.cgi?type=PEPDOCK). This study presents an example of GalexyPepDock performed by each epitope and available PDB file of HLA alleles, separately.

### Vaccine construction

To construct effectual vaccine components, we fused the antigenic epitopes with the help of specific peptide linkers. Three different constructs for each linear B lymphocyte (LBL), cytotoxic T lymphocyte (CTL) and helper T lymphocyte (HTL) were designed.

### The physicochemical parameters

The physicochemical properties of the designed LBL, CTL and HTL epitopes including molecular weight, theoretical PI, positive and negative charge residue, solubility and stability were evaluated by ProtParam online server (http://us.expasy.org/tools/protparam.html) [29].

### 3D structure prediction

I-TASSER server [30] (https://zhanglab.ccmb.med.umich.edu/I-TASSER/) was used for modeling the 3D structure of designed constructs. This server is in active development with the goal to provide the most accurate protein structure and function predictions using state-of-the-art algorithms. After analysis, the models with the highest confidence score (C-score) were selected for refinement analysis.

### Refinement and validation of tertiary structure

GalaxyRefine 2 Server [31] (http://galaxy.seoklab.org/cgi-bin/submit.cgi?type=REFINE2) was used to refine predicted tertiary structures. GalaxyRefine2 performs iterative optimization with several geometric operators to increase the accuracy of the initial models. Final Refined models were analyzed by SAVE5.0 (https://servicesn.mbi.ucla.edu/SAVES/) server to validated tertiary structures. SAVE server gives Ramachandran plot of the whole structure, determines the overall quality of tertiary structure, and calculates buried protein atoms, stereochemical quality and atomic interaction of predicted 3D structure.

### Discontinuous B-cell epitope prediction

Prediction of discontinuous B-cell epitope needs tertiary structure of a protein or polypeptide since the interaction between antigen epitopes and antibodies is very important. As regards, after refinement and validation analysis, the 3D structure of constructs were assessed by the Ellipro server [32] (https://tools.iedb.org/ellipro/help/) to find discontinuous B-cell epitopes. ElliPro web-based server uses modified Thornton’s method along with residue clustering algorithms. In this study, epitope prediction parameters (minimum score and maximum distance) were set to default values (0.5 and 6).

### Docking between vaccine constructs and Toll-Like Receptors (TLRs)

TLRs are sensors recognizing molecular patterns of pathogens to initiate innate immune system. It was demonstrated that TLRs 2, 3 and 4 are more susceptible to Coronaviridae family including SARS-CoV and MERS-CoV [33–35]. Thus, PDB files of TLRs 2, 3 and 4 were obtained from Protein Data Bank (http://www.rcsb.org/) and then protein-protein docking between three vaccine constructs and TLRs were performed by ClusPro server [36] (https://cluspro.bu.edu/). ClusPro uses three steps algorithms containing 1) Rigid-body docking, 2) Cluster retained conformations, and 3) Refine by CHARMM minimization.

## Results

### The sequences of the structural SARS-CoV-2 proteins

The reference sequences of the structural proteins (S, N and M, NC_045512.2) were obtained from NCBI. The sequence was downloaded in a FASTA format to carry out further analyses.

### Prediction of linear B-cell epitopes

We obtained a total of 44 sequential linear B-cell epitopes with variable lengths from IEDB server within three main proteins of SARS-CoV-2 (*i*.*e*., S, N and M), and the ability of epitopes in inducing different classes of antibody in IgPred server were analyzed. In S protein, S^1133-1172^ (VNNTVYDPLQPELDSFKEELDKYFKNHTSPDVDLGDISGI), S^440-501^ (NLDSKVGGNYNYLYRLFRKSNLKPFERDISTEIYQAGSTPCNGVEGFNCYFPLQSYGFQPTN), S^59-81^(FSNVTWFHAIHVSGTNGTKRFDN) and S^304-322^ (KSFTVEKGIYQTSNFRVQP), and in N protein, N^232-269^(SKMSGKGQQQQGQTVTKKSAAEASKKPRQKRTATKAYN), N^164-216^(GTTLPKGFYAEGSRGGSQASSRSSSRSRNSSRNSTPGSSRGTSPARMAGNGGD), N^1-51^(MSDNGPQNQRNAPRITFGGPSDSTGSNQNGERSGARSKQRRPQGLPNNTAS) and N^361-390^(KTFPPTEPKKDKKKKADETQALPQRQKKQQ) were chosen as they had the ability to induce antibody (Table 1). In case of M protein, we found three epitopes. However, we ruled out M epitope for potential B-cell epitope as they were unable to induce any class of the antibodies.

**Table 1:** The selected LBL* epitopes of SARS-CoV-2 based on binding affinity.

Protein Name	Position	Epitope Sequence	Antibody Class Prediction
S	1133–1172	VNNTVYDPLQPELDSFKEELDKYFKNHTSPDVDLGDISGI	IgG
	440–501	NLDSKVGGNYNYLYRLFRKSNLKPFERDISTEIYQAGSTPCNGVEGFNCYFPLQSYGFQPTN	IgG
	59–81	FSNVTWFHAIHVSGTNGTKRFDN	IgG
	304–322	KSFTVEKGIYQTSNFRVQP	IgA
N	232–269	SKMSGKGQQQQGQTVTKKSAAEASKKPRQKRTATKAYN	IgG
	164–216	GTTLPKGFYAEGSRGGSQASSRSSSRSRNSSRNSTPGSSRGTSPARMAGNGGD	IgG
	1–51	MSDNGPQNQRNAPRITFGGPSDSTGSNQNGERSGARSKQRRPQGLPNNTAS	IgG
	361–390	KTFPPTEPKKDKKKKADETQALPQRQKKQQ	IgG

*Linear B lymphocyte

### Prediction of T-cell epitopes

Identification of CD8^+^ cytotoxic T lymphocyte (CTL) epitopes is a crucial step in epitope-driven vaccine design as MHC class I restricted CTL plays a critical role in controlling viral infections. In this study, we employed NetMHCpan and NetMHCIIpan as mentioned procedure in below.

### MHC class I prediction

The SARS-CoV-2 protein sequences were analyzed by NetMHCpan 4.1 server to identify the most immunodominant regions. In each protein, peptides with the highest binding affinity scores were determined as high-potential CTL epitope candidates. In each protein, the best epitopes with higher binding affinity were selected as the putative CTL epitope based on calculated average immunogenicity scores. Chosen MHC-I epitopes were listed in Table 2 with encountering MHC alleles, average rank scores, conservancy prediction and allergenicity assessment. Also, all of the chosen sequences of epitopes were non-allergen and 100% conserved within four clades.

**Table 2:** The selected CTL* epitopes of SARS-CoV-2 based on binding affinity.

Protein Name	Position	Epitope Sequence	No. of Alleles	NetMHCpan Average Rank Scores**	Conservancy	Allergenicity
S	27–37	YTNSFTRGVYY	12	0.67	S:100%*** V: 100% G: 100% O: 100%	Non-allergen
	686–696	VASQSIIAYTM	12	0.69	S:100% V: 100% G: 100% O: 100%	Non-allergen
	191–199	FVFKNIDGY	10	0.66	S:100% V: 100% G: 100% O: 100%	Non-allergen
	1051–1061	FPQSAPHGVVF	10	1.08	S:100% V: 100% G: 100% O: 100%	Non-allergen
	257–265	WTAGAAAYY	9	0.44	S:100% V: 100% G: 100% O: 100%	Non-allergen
	603–611	TSNQVAVLY	9	0.44	S:100% V: 100% G: 100% O: 100%	Non-allergen
	868–876	MIAQYTSAL	9	0.61	S:100% V: 100% G: 100% O: 100%	Non-allergen
	161–169	SANNCTFEY	8	0.96	S:100% V: 100% G: 100% O: 100%	Non-allergen
	360–368	CVADYSVLY	8	0.48	S:100% V: 100% G: 100% O: 100%	Non-allergen
	1094–1102	FVSNGTHWF	8	0.69	S:100% V: 100% G: 100% O: 100%	Non-allergen
N	103–113	LSPRWYFYYLG	10	1.04	S:100% V: 100% G: 100% O: 100%	Non-allergen
	304–313	AQFAPSASAF	8	0.87	S:100% V: 100% G: 100% O: 100%	Non-allergen
	47–55	NTASWFTAL	6	0.70	S:100% V: 100% G: 100% O: 100%	Non-allergen
	264–273	TKAYNVTQAF	6	0.90	S:100% V: 100% G: 100% O: 100%	Non-allergen
	52–60	FTALTQHGK	5	0.64	S:100% V: 100% G: 100% O: 100%	Non-allergen
M	36–46	FAYANRNRFLY	13	1.23	S:100% V: 100% G: 100% O: 100%	Non-allergen
	168–180	TVATSRTLSYY	9	0.89	S:100% V: 100% G: 100% O: 100%	Non-allergen
	195–204	YSRYRIGNYK	9	0.31	S:100% V: 100% G: 100% O: 100%	Non-allergen
	90–99	MWLSYFIASF	7	1.05	S:100% V: 100% G: 100% O: 100%	Non-allergen
	102–111	FARTRSMWSF	7	0.59	S:100% V: 100% G: 100% O: 100%	Non-allergen

* Cytotoxic T lymphocyte

** Lower rates show better binding affinity

*** There are four clades of SARS-CoV-2 according to GISAID database

### MHC class II prediction

The SARS-CoV-2 protein sequences were analyzed by NetMHCIIpan 4.0 server to identify MHC-II epitope. Epitopes with the maximum number of binding HLA-DR alleles were selected as putative HTL epitope candidate. Chosen MHC-II epitopes were listed in Table 3 with encountering MHC alleles, average rank scores, conservancy and antibody-specific epitopes prediction, and allergenicity assessment. Also, all of the chosen sequences of epitopes were non-allergen and 100% conserved within four clades.

**Table 3:** The selected HTL epitopes of SARS-CoV-2 based on binding affinity.

Protein Name	Position	Epitope Sequence	No. of Alleles	NetMHCpan Average Rank Scores*	Conservancy	Allergenicity	IFN-gamma prediction	IL-10 prediction
S	196–231	NIDGYFKIYSKHTPINLVRDLPQGFS	25	4.29	S:100% V: 100% G: 100% O: 100%	Non-allergen	Positive	Positive
	303–323	LKSFTVEKGIYQTSNFRVQPT	25	3.96	S:100% V: 100% G: 100% O: 100%	Non-allergen	Positive	Negative
	313–330	YQTSNFRVQPTESIVRFP	25	3.17	S:100% V: 100% G: 100% O: 100%	Non-allergen	Positive	Positive
	1009–1030	TQQLIRAAEIRASANLAATKMS	25	3.89	S:100% V: 100% G: 100% O: 100%	Non-allergen	Positive	Positive
	32–53	FTRGVYYPDKVFRSSVLHSTQD	24	4.45	S:100% V: 100% G: 100% O: 100%	Non-allergen	Positive	Positive
	1057–1074	PHGVVFLHVTYVPAQEKN	22	5.7	S:100% V: 100% G: 100% O: 100%	Non-allergen	Positive	Positive
	689–704	SQSIIAYTMSLGAENS	21	5.2	S:100% V: 100% G: 100% O: 100%	Non-allergen	Positive	Positive
	801–817	NFSQILPDPSKPSKRSF	20	3.51	S:100% V: 100% G: 100% O: 100%	Non-allergen	Negative	Positive
	1110–1126	YEPQIITTDNTFVSGNC	19	3.19	S:100% V: 100% G: 100% O: 100%	Non-allergen	Negative	Positive
	114–130	TQSLLIVNNATNVVIKV	19	5.37	S:100% V: 100% G: 100% O: 100%	Non-allergen	Positive	Positive
N	328–349	GTWLTYTGAIKLDDKDPNFKDQ	25	4.5	S:100% V: 100% G: 100% O: 100%	Non-allergen	Positive	Negative
	126–143	NKDGIIWVATEGALNTPK	24	3.85	S:100% V: 100% G: 100% O: 100%	Non-allergen	Positive	Negative
	342–361	KDPNFKDQVILLNKHIDAYK	24	4.39	S:100% V: 100% G: 100% O: 100%	Non-allergen	Positive	Positive
	48–63	NTASWFTALTQHGKED	23	3.24	S:100% V: 100% G: 100% O: 100%	Non-allergen	Positive	Negative
	167–181	LPKGFYAEGSRGGSQ	23	2	S:100% V: 100% G: 100% O: 100%	Non-allergen	Negative	Negative
	405–419	KQLQQSMSSADSTQA	22	3.58	S:100% V: 100% G: 100% O: 100%	Non-allergen	Negative	Negative
M	198–217	RYRIGNYKLNTDHSSSSDNI	22	4.76	S:100% V: 100% G: 100% O: 100%	Non-allergen	Positive	Negative
	173–191	SRTLSYYKLGASQRVAGDS	20	4.53	S:100% V: 100% G: 100% O: 100%	Non-allergen	Positive	Negative
	107–128	RSMWSFNPETNILLNVPLHGTI	19	6.02	S:100% V: 100% G: 100% O: 100%	Non-allergen	Positive	Negative
	163–181	DLPKEITVATSRTLSYYKL	17	3.57	S:100% V: 100% G: 100% O: 100%	Non-allergen	Positive	Negative

*lower rates show better binding affinity

### Tap transport/proteasomal cleavage

Tap transport and proteasomal cleavage are as important as binding affinity in antigen presentation pathway to CTLs. In this case, NetCTL1.2 server was used. All of the epitopes shown in Table 4 have upper cut off identification scores (> 0.75) which show a high quality of proteasomal cleavage and Tap transport efficiency. Among all epitopes, S^257-265^ and S^603-611^ have the highest epitope identification score of 3.14 and 3.07, respectively.

**Table 4:** Proteasomal cleavage and TAP transport efficiency scores of MHC-I predicted epitopes.

Protein Name	Position	Epitope Sequence	Proteasomal C -terminal cleavage Score*	TAP transport efficiency Score**	Epitope identification Score***
**S**	27–37	YTNSFTRGVYY	1.43	0.96	1.73
	686–696	VASQSIIAYTM	1.49	0.93	1.73
	191–199	FVFKNIDGY	1.75	0.94	2.03
	1051–1061	FPQSAPHGVVF	0.98	0.93	1.18
	257–265	WTAGAAAYY	2.85	0.94	3.14
	603–611	TSNQVAVLY	2.87	0.95	3.07
	868–876	MIAQYTSAL	1.04	0.93	1.33
	161–169	SANNCTFEY	2.07	0.92	2.36
	360–368	CVADYSVLY	2.27	0.97	2.56
	1094–1102	FVSNGTHWF	1.46	0.86	1.72
**N**	103–113	LSPRWYFYYLG	2.05	0.96	2.34
	304–313	AQFAPSASAF	1.06	0.66	1.31
	47–55	NTASWFTAL	1.08	0.94	1.27
	264–273	TKAYNVTQAF	1.01	0.94	1.30
	52–60	FTALTQHGK	0.80	0.72	0.92
**M**	36–46	FAYANRNRFLY	1.40	0.95	1.69
	168–180	TVATSRTLSYY	2.31	0.96	2.61
	195–204	YSRYRIGNYK	1.36	0.84	1.64
	90–99	MWLSYFIASF	0.66	0.97	0.96
	102–111	FARTRSMWSF	1.10	0.95	1.38

*Higher rates show better quality of proteasomal cleavage

** Higher rates show better quality of tap transport efficiency

*** Higher rates show better quality of epitope identification

### Population coverage

As mentioned above, MHC polymorphisms are dramatically at different frequencies in various ethnicities. Thus, careful consideration should be given to the way of effective vaccine development. In this study, population coverage was estimated separately for each putative epitope in different geographical regions (Tables 5 and 6). For CTL epitopes, the highest population coverage of the world’s population was calculated for S^27-37^ with 86.27%. For helper T-cell epitopes, the highest population coverage of the world’s population was calculated for S^196-231^, S^303-323^, S^313-330^, S^1009-1030^ and N^328-349^ with 90.33%.

**Table 5:** Population coverage of putative SARS-CoV-2 CTL epitopes.

Area	S^27-37^	S^686-696^	S^191-199^	S^1051-1061^	S^257-265^	S^603-611^	S^868-876^	S^161-169^	S^360-368^	S^1094-1102^	N^103-113^	N^304-313^	N^47-55^	N^264-273^	N^52-60^	M^36-46^	M^168-180^	M^195-204^	M^90-99^	M^102-111^
**Central Africa**	58.3%	61.19%	47.7%	42.84%	45.43%	45.43%	47.79%	41.35%	41.35%	37.02%	39.4%	33.9%	31.58%	38.38%	27.68%	46.0%	43.11%	34.13%	25.22%	30.74%
**East Africa**	69.68%	67.18%	54.97%	51.17%	50.59%	50.59%	54.01%	44.19%	44.19%	48.33%	47.25%	27.24%	27.26%	29.76%	31.08%	53.0%	48.86%	41.54%	28.22%	28.53%
**East Asia**	68.17%	59.86%	55.61%	54.86%	55.61%	55.61%	60.36%	55.49%	53.53%	74.58%	78.7%	82.83%	31.06%	70.63%	32.55%	84.27%	54.94%	45.63%	71.67%	35.38%
**Europe**	93.95%	80.28%	77.35%	71.73%	73.88%	73.88%	79.54%	73.47%	73.45%	61.27%	76.7%	71.17%	52.65%	64.39%	64.85%	85.33%	70.88%	70.14%	59.55%	50.55%
**North Africa**	76.03%	73.45%	57.9%	50.94%	55.03%	55.03%	59.15%	52.23%	52.0%	52.87%	55.62%	44.7%	30.4%	34.85%	36.01%	57.8%	50.85%	46.2%	37.72%	29.94%
**North America**	84.8%	69.76%	64.08%	57.57%	61.39%	61.39%	70.66%	59.51%	59.28%	57.58%	69.4%	67.35%	38.65%	57.07%	44.89%	75.68%	57.57%	52.73%	51.7%	40.09%
**Northeast Asia**	73.97%	68.15%	66.77%	37.1%	66.51%	66.51%	38.22%	63.48%	63.43%	50.44%	73.37%	77.42%	13.17%	46.14%	52.93%	76.81%	65.94%	61.92%	41.39%	18.03%
**Oceania**	63.86%	58.32%	54.78%	34.54%	54.21%	54.21%	31.12%	52.96%	52.96%	66.01%	84.26%	83.22%	21.79%	59.46%	50.2%	86.64%	52.61%	51.88%	60.32%	17.61%
**South Africa**	64.94%	72.02%	57.59%	49.1%	55.81%	55.81%	44.82%	53.25%	53.25%	50.83%	58.64%	46.87%	28.43%	33.15%	41.9%	60.92%	50.64%	50.62%	37.25%	29.34%
**South America**	56.97%	45.93%	40.8%	25.13%	37.66%	37.66%	36.75%	36.75%	36.75%	39.03%	44.64%	52.03%	16.62%	37.72%	31.08%	57.06%	27.16%	34.24%	36.7%	14.41%
**South Asia**	77.05%	73.24%	71.64%	46.92%	70.83%	70.83%	38.84%	67.75%	67.75%	54.67%	67.42%	66.54%	29.6%	43.15%	59.67%	72.8%	55.62%	65.66%	37.59%	27.3%
**Southeast Asia**	66.67%	62.24%	58.2%	35.31%	55.92%	55.92%	28.83%	48.97%	48.97%	57.94%	77.29%	82.1%	17.1%	53.25%	43.57%	80.89%	52.67%	47.52%	51.06%	21.82%
**Southwest Asia**	77.27%	66.35%	59.6%	45.9%	56.9%	56.9%	54.88%	54.49%	54.49%	48.92%	58.54%	51.82%	29.33%	32.89%	47.47%	63.53%	51.56%	51.97%	38.63%	29.55%
**West Africa**	70.28%	74.43%	58.96%	53.85%	56.47%	56.47%	57.29%	51.92%	51.92%	48.96%	55.22%	47.07%	38.36%	43.67%	33.19%	61.05%	55.9%	41.03%	41.74%	42.38%
**West Indies**	80.64%	74.78%	63.01%	56.85%	59.44%	59.44%	64.77%	56.07%	56.07%	54.84%	64.26%	61.14%	41.35%	54.32%	47.92%	73.47%	55.9%	50.81%	49.62%	42.73%
**World**	**86.27%**	**72.24%**	**68.17%**	**57.5%**	**65.34%**	**65.34%**	**67.31%**	**64.18%**	**64.0%**	**56.47%**	**70.23%**	**68.46%**	**39.15%**	**55.99%**	**54.27%**	**77.72%**	**62.15%**	**60.54%**	**50.99%**	**36.77%**

**Table 6:** Population coverage of putative SARS-CoV-2 HTL epitopes.

Area	S^196-231^	S^303-323^	S^313-330^	S^1009-1030^	S^32-53^	S^1057-1074^	S^689-704^	S^801-817^	S^1110-1126^	S^114-130^	N^328-349^	N^126-143^	N^342-361^	N^48-63^	N^167-181^	N^405-419^	M^198-217^	M^173-191^	M^107-128^	M^163-181^
**Central Africa**	75.33%	75.33%	75.33%	75.33%	71.52%	59.02%	59.34%	60.66%	64.38%	66.12%	75.33%	67.04%	74.78%	66.93%	64.03%	56.8%	61.37%	70.37%	59.27%	65.04%
**East Africa**	81.34%	81.34%	81.34%	81.34%	76.57%	65.54%	58.27%	68.08%	62.42%	65.07%	81.34%	73.27%	80.55%	74.6%	69.31%	56.04%	65.89%	74.4%	62.42%	64.24%
**East Asia**	86.93%	86.93%	86.93%	86.93%	84.15%	84.31%	81.62%	62.84%	79.63%	73.29%	86.93%	86.13%	80.46%	79.92%	82.21%	77.93%	72.87%	72.62%	67.69%	66.34%
**Europe**	93.54%	93.54%	93.54%	93.54%	92.38%	81.75%	78.89%	72.95%	86.86%	87.15%	93.54%	90.14%	93.42%	77.57%	84.85%	76.43%	87.88%	91.26%	80.17%	81.71%
**North Africa**	88.26%	88.26%	88.26%	88.26%	87.65%	74.7%	72.59%	73.18%	80.37%	82.82%	88.26%	85.14%	87.95%	71.69%	78.01%	75.59%	83.78%	83.49%	68.5%	76.74%
**North America**	94.75%	94.75%	94.75%	94.75%	93.79%	84.42%	82.17%	77.86%	88.13%	88.84%	94.75%	92.18%	94.5%	73.57%	86.73%	79.13%	89.33%	93.19%	83.03%	83.13%
**Northeast Asia**	68.85%	68.85%	68.85%	68.85%	65.35%	63.16%	60.58%	47.01%	62.19%	54.36%	68.85%	66.94%	65.74%	78.32%	61.48%	54.28%	53.05%	59.87%	54.07%	56.44%
**Oceania**	74.82%	74.82%	74.82%	74.82%	69.27%	73.93%	62.41%	62.75%	61.98%	58.06%	74.82%	74.55%	68.85%	56.81%	68.59%	54.01%	64.28%	53.78%	52.62%	43.72%
**South Africa**	52.11%	52.11%	52.11%	52.11%	52.11%	8.42%	31.28%	50.86%	51.56%	50.86%	52.11%	32.76%	52.11%	66.55%	31.28%	32.76%	31.28%	52.11%	30.61%	52.11%
**South America**	76.19%	76.19%	76.19%	76.19%	75.35%	69.22%	68.04%	62.65%	58.82%	68.62%	76.19%	73.99%	67.94%	31.28%	70.7%	68.41%	68.85%	71.95%	65.09%	54.89%
**South Asia**	88.43%	88.43%	88.43%	88.43%	87.25%	77.38%	75.43%	66.62%	77.06%	82.25%	88.43%	84.78%	88.0%	68.74%	80.41%	71.04%	82.52%	85.41%	71.07%	79.51%
**Southeast Asia**	67.66%	67.66%	67.66%	67.66%	65.26%	62.42%	56.06%	50.07%	59.47%	50.67%	67.66%	67.25%	62.15%	73.82%	60.3%	53.94%	54.69%	56.91%	49.7%	51.81%
**Southwest Asia**	58.07%	58.07%	58.07%	58.07%	57.31%	48.2%	44.41%	45.91%	48.46%	50.87%	58.07%	54.83%	57.15%	56.04%	50.87%	45.49%	53.64%	52.81%	43.09%	43.57%
**West Africa**	84.27%	84.27%	84.27%	84.27%	83.28%	75.29%	72.9%	71.93%	64.44%	72.66%	84.27%	79.83%	83.27%	48.87%	79.11%	70.05%	73.8%	78.54%	60.66%	69.82%
**West Indies**	77.4%	77.4%	77.4%	77.4%	74.21%	69.63%	62.33%	52.95%	64.29%	64.15%	77.4%	73.64%	76.45%	77.74%	70.24%	59.75%	67.69%	72.67%	65.6%	61.47%
**World**	**90.33%**	**90.33%**	**90.33%**	**90.33%**	**88.87%**	**79.8%**	**77.08%**	**70.7%**	**82.52%**	**82.55%**	**90.33%**	**87.34%**	**89.71%**	**74.87%**	**81.75%**	**74.33%**	**83.11%**	**87.1%**	**76.39%**	**76.94%**

### Peptide-protein flexible docking

At first, available structure data of MHC-I and MHC-II were downloaded from RCSB PDB server (https://www.rcsb.org/). All potential epitopes and MHC PDB files were submitted to the server separately. Then, top models with the highest interaction similarity score (similarity of the amino acids of the target complex aligned to the contacting residues in the template structure to the template amino acids, obtained from GalexyPepDock server) were selected for each peptide and its MHC. For CTL epitopes, N^103-113^, S^868-876^, M^36-46^, S^1094-1102^, M^102-111^, S^1051-1061^, S^360-368^, S^191-199^ and S^686-696^ had the highest average of interaction similarity score, respectively (Table 7). For HTL epitopes, M^107-128^, N^48-63^, S^689-704^, S^1057-1074^, S^196-231^, N^328-349^, S^32-53^, N^126-143^, M^163-181^ and S^114-130^ had the highest average of interaction similarity score, respectively (Table 8). Overall, CTL epitopes showed better quality of docking in comparison with HTL epitope.

**Table 7:** Interaction similarity scores of the selected CTL epitopes using GalexyPepDock flexible docking server.

Protein	Epitope	HLA A0101	HLA A0201	HLA A0301	HLA A2402	HLA A1101	HLA B0702	HLA B0801	HLA B2705	HLA B3501	HLA B5101	Average*
**S**	**YTNSFTRGVYY**	170	192	184	172	181	186	199	175	219	202	188.0
	**SANNCTFEY**	195	170	153	160	158	171	167	168	213	185	174.0
	**FVFKNIDGY**	188	209	195	187	197	219	237	206	214	191	204.3
	**WTAGAAAYY**	151	181	165	178	159	170	189	176	201	190	176.0
	**CVADYSVLY**	203	231	223	190	216	201	192	207	232	212	210.7
	**TSNQVAVLY**	157	163	152	170	151	170	158	171	216	188	169.6
	**VASQSIIAYTM**	174	201	187	206	200	203	199	195	242	225	203.2
	**MIAQYTSAL**	205	266	239	237	220	219	227	219	229	225	228.6
	**FPQSAPHGVVF**	177	216	189	204	179	215	243	200	227	258	210.8
	**FVSNGTHWF**	199	207	189	217	208	235	256	221	220	201	215.3
**M**	**FAYANRNRFLY**	210	232	202	268	213	213	236	211	253	226	226.4
	**MWLSYFIASF**	171	231	204	219	185	197	214	187	191	195	199.4
	**FARTRSMWSF**	195	201	196	260	198	211	249	204	200	215	212.9
	**TVATSRTLSYY**	164	201	185	191	180	209	188	207	251	223	199.9
	**YSRYRIGNYK**	154	176	171	215	170	180	215	178	219	206	188.4
**N**	**NTASWFTAL**	153	189	168	192	174	189	177	185	187	181	179.5
	**FTALTQHGK**	167	186	166	170	176	206	232	191	188	176	185.8
	**LSPRWYFYYLG**	217	244	237	204	237	262	247	254	300	278	248
	**TKAYNVTQAF**	142	186	151	207	160	168	158	174	179	200	172.5
	**AQFAPSASAF**	172	201	192	203	188	195	192	191	213	215	196.2

* Higher rates show better quality of modeling

**Table 8:** Interaction similarity scores of the selected HTL epitopes using GalexyPepDock flexible docking server.

Protein	Epitope	DRB1-0101	DRB1-0301	DRB1-0401	DRB1-1101	DRB1- 1501	DRB5-0101	Average*
S	**NIDGYFKIYSKHTPINLVRDLPQGFS**	139	146	132	132	132	132	135.5
	**LKSFTVEKGIYQTSNFRVQPT**	124	126	124	126	124	126	125.0
	**YQTSNFRVQPTESIVRFP**	118	105	118	118	108	118	114.1
	**TQQLIRAAEIRASANLAATKMS**	133	123	113	123	113	113	119.6
	**FTRGVYYPDKVFRSSVLHSTQD**	135	133	135	133	135	135	134.3
	**PHGVVFLHVTYVPAQEKN**	170	128	128	128	132	128	135.6
	**SQSIIAYTMSLGAENS**	141	129	141	141	141	141	139.0
	**NFSQILPDPSKPSKRSF**	148	126	126	126	126	126	129.6
	**YEPQIITTDNTFVSGNC**	123	116	120	116	123	120	119.6
	**TQSLLIVNNATNVVIKV**	131	131	131	130	130	131	130.6
M	**RYRIGNYKLNTDHSSSSDNI**	117	113	117	117	117	117	116.3
	**SRTLSYYKLGASQRVAGDS**	124	124	124	128	124	128	125.3
	**RSMWSFNPETNILLNVPLHGTI**	169	169	169	139	169	169	164.0
	**DLPKEITVATSRTLSYYKL**	137	124	125	137	137	137	132.8
N	**GTWLTYTGAIKLDDKDPNFKDQ**	137	131	137	137	137	131	135
	**NKDGIIWVATEGALNTPK**	136	133	131	136	131	136	133.8
	**KDPNFKDQVILLNKHIDAYK**	127	134	127	127	134	127	129.3
	**NTASWFTALTQHGKED**	137	137	137	137	149	137	139.0
	**LPKGFYAEGSRGGSQ**	107	107	107	105	105	107	106.3
	**KQLQQSMSSADSTQA**	142	118	118	118	118	118	122.0

* Higher rates show better quality of modeling

### Construct design

**Fig 1:**
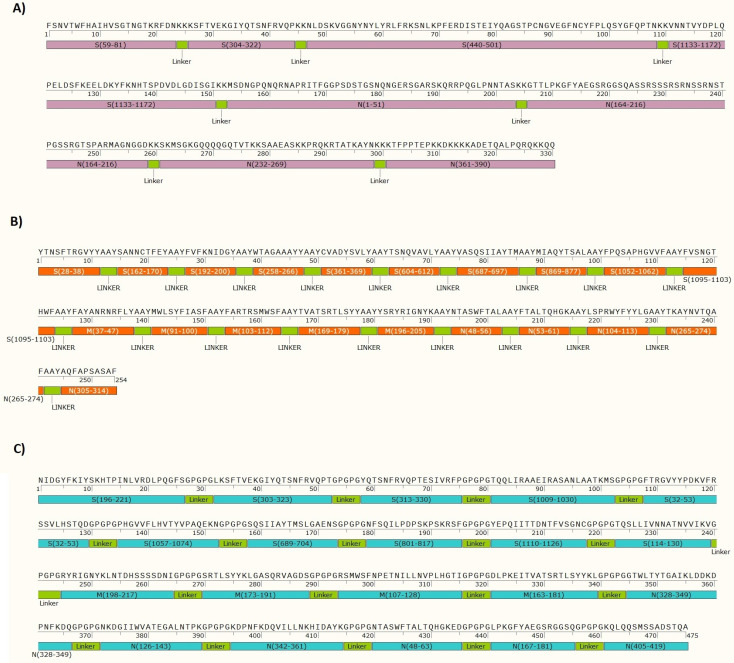
The suggested epitope constructs: a) the schematic diagram of the LBL epitope construct derived from the S and N proteins of the SARS-CoV-2 linked by KK linker; b) the schematic diagram of the CTL epitope construct derived from the S, M and N proteins of the SARS-CoV-2 linked by AAY linker; c) the schematic diagram of the HTL epitope construct derived from the S, M and N proteins of the SARS-CoV-2 linked by GPGPG linker.

According to above-mentioned parameters including binding affinity between peptide and MHCs, epitope identification scores for T-cells, antibody-specific epitopes prediction for B-cells and T-cells, proteasomal cleavage and Tap transport scores, allergenicity, conservancy degree, population coverage and peptide-protein flexible docking scores, three different constructs were designed by top-ranked epitopes (Fig 1). For B-cell structure, S^1133-1172^, S^440-501^, S^59-81^, S^304-322^, N^232-269^, N^164-216^, N^1-51^ and N^361-390^ epitopes had the ability to induce antibodies. The B-cell epitopes were linked by KK linker [37]. For CTL structure, S^27-37^, S^161-169^, S^191-199^, S^257-265^, S^360-368^, S^603-611^, S^686-696^, S^868-876^, S^1051-1061^, S^1094-1102^, M^36-46^, M^90-99^, M^102-111^, M^195-204^, N^47-55^, N^52-60^, N^103-113^, N^264-273^ and N^304-313^ were selected. The CTL epitopes were linked by AAY linker [37], and finally, for Helper T-cell (HTL) structure, S^32-53^, S^114-130^, S^196-231^, S^303-323^, S^313-330^, S^689-704^, S^801-817^, S^1009-1030^, S^1057-1074^, S^1110-1126^, M^107-128^, M^163-181^, M^173-191^, M^198-217^, N^48-63^, N^126-143^, N^167-181^, N^328-349^, N^342-361^ and N^405^ were selected. The HTL epitopes were linked by GPGPG linker [37].

### The physicochemical parameters

Three constructs for each LBL, CTL and HTL epitope were analyzed by ProtParam server. Physicochemical properties of the constructed peptides were shown in Table 9. For LBL epitope, molecular weight (MW) was measured 36.5 kDa with theoretical isoelectric point (PI) of 10.24. For CTL and HTL epitopes, MWs were measured 28.6 and 49.5 kDa with PIs of 9.29 and 9.42, respectively. All constructs were soluble and stable.

**Table 9:** Physicochemical properties of the designed HTL and CTL and LBL epitopes.

Construct	Molecular weight	Theoretical PI	Positive charge residue	Negative charge residue	Solubility	Stability
LBL-epitope	36.5 kDa	10.24	63	26	Soluble	Stable
CTL-epitope	28.6 kDa	9.29	13	3	Soluble	Stable
HTL-epitope	49.5 kDa	9.42	42	31	Soluble	Stable

### 3D structure prediction

**Fig 2:**
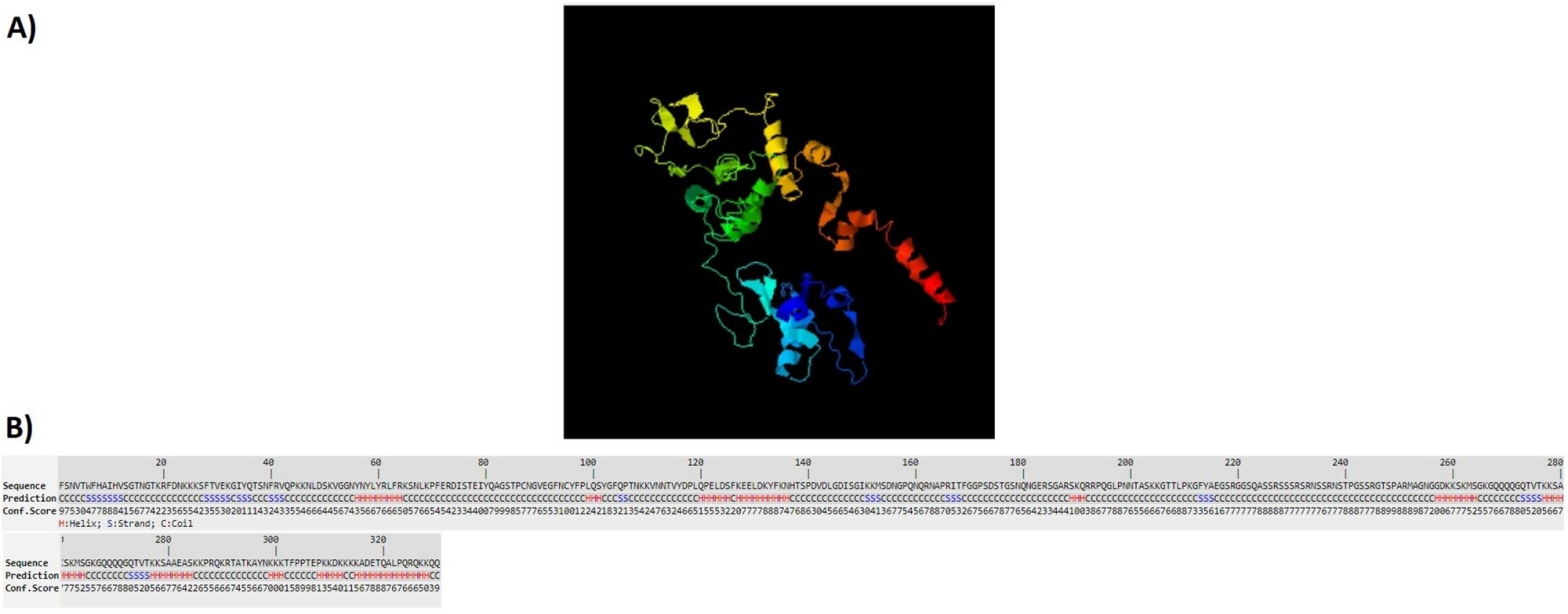
The tertiary structure of the predicted LBL construct: a) 3D prediction of LBL multiepitope construct, b) sequence prediction and conformation scores of each amino acid in LBL multiepitope construct.

**Fig 3:**
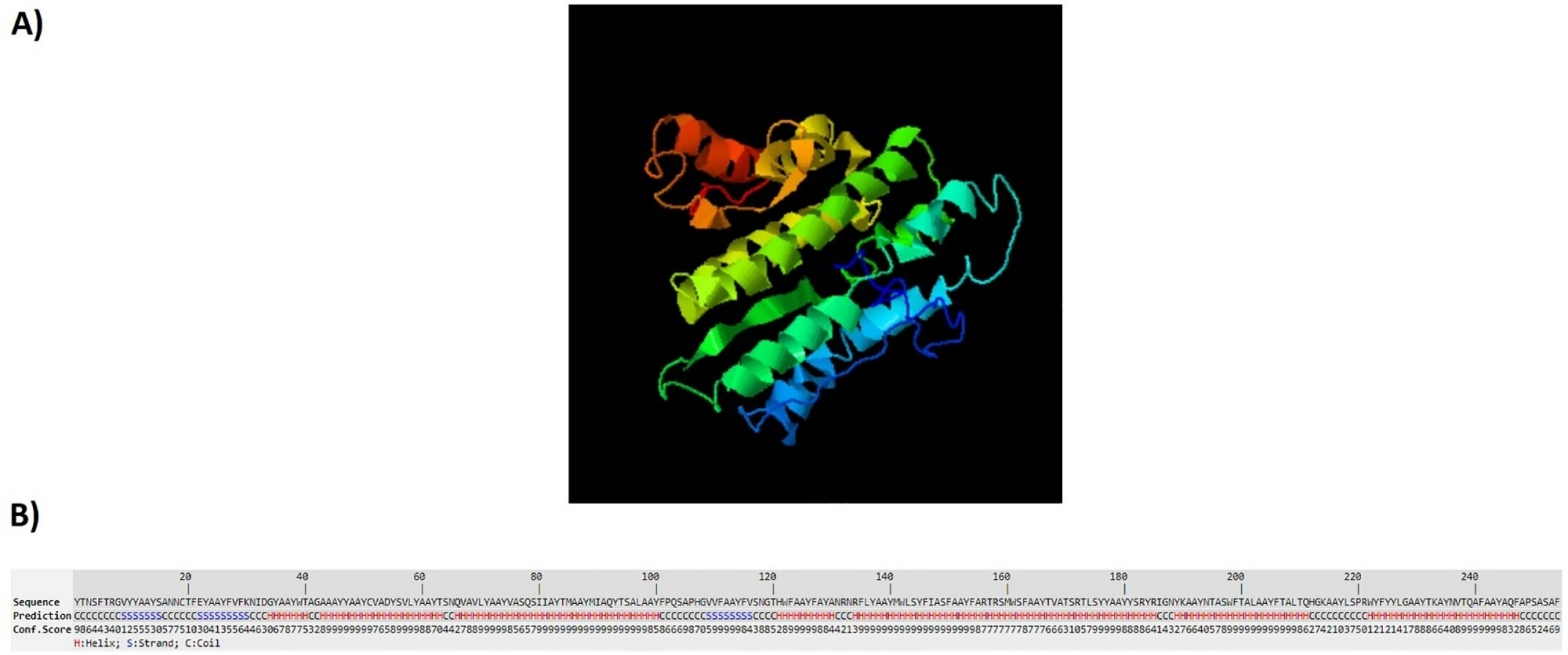
The tertiary structure of the predicted CTL construct: a) 3D prediction of CTL multi-epitope construct, b) sequence prediction and conformation scores of each amino acid in CTL multiepitope construct.

**Fig 4:**
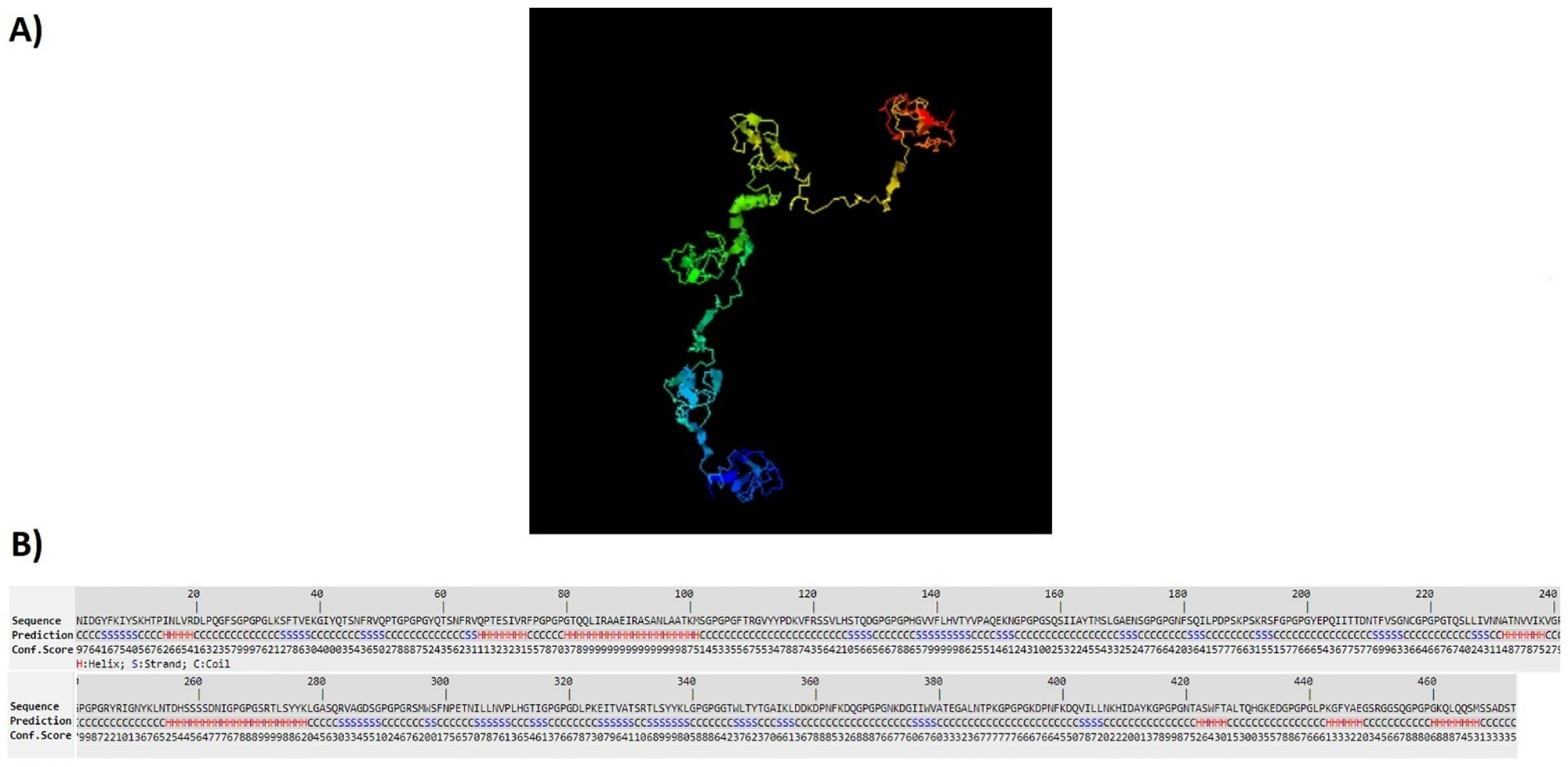
The tertiary structure of the predicted HTL construct: a) 3D prediction of HTL multiepitope construct, b) sequence prediction and conformation scores of each amino acid in HTL multiepitope construct.

The designed structures were analyzed by I-TASSER server. This server generates some structural conformations, then uses SPICKER program to cluster all structures based on the pair-wise structure similarity. Finally, the top five models corresponding to the five largest clusters were reported by the server. The assurance of each model was calculated by C-score. The C-score values show the accuracy of the predicted model which usually is in the range of -5 to 2. Also, the higher value of the C-score signifies the better quality of prediction. The C-scores of the models for LBL, CTL and HTL polypeptide constructs were -2.39, -4.42 and -0.63, respectively. Figs 2–4 show tertiary structures of the predicted LBL, CTL and HTL structures.

### Refinement and validation of 3D structures

**Fig 5:**
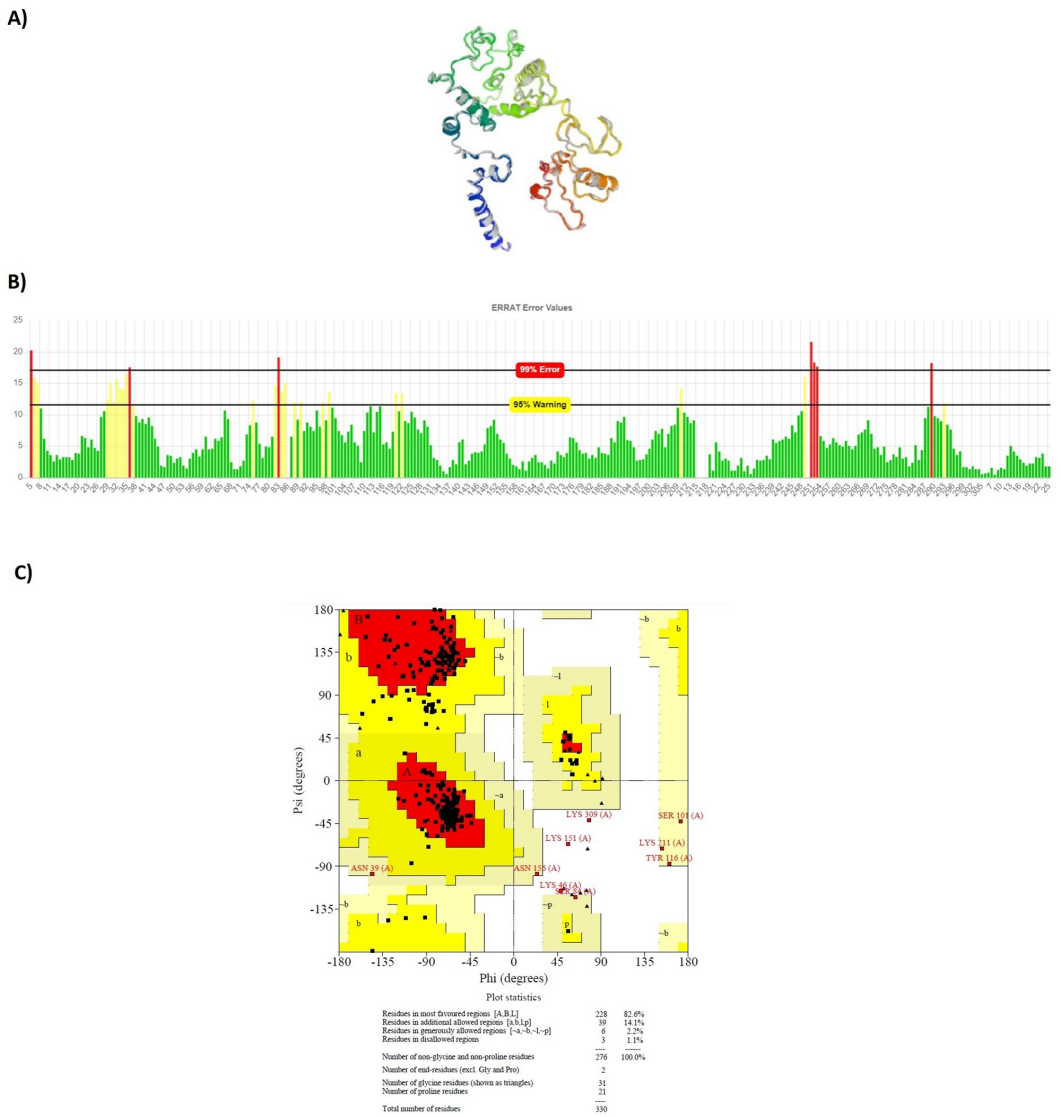
Refined characteristics of LBL multiepitope construct: a) Refined 3D prediction of LBL multiepitope construct, b) overall quality of refinement, c) Ramachandran plot.

**Fig 6:**
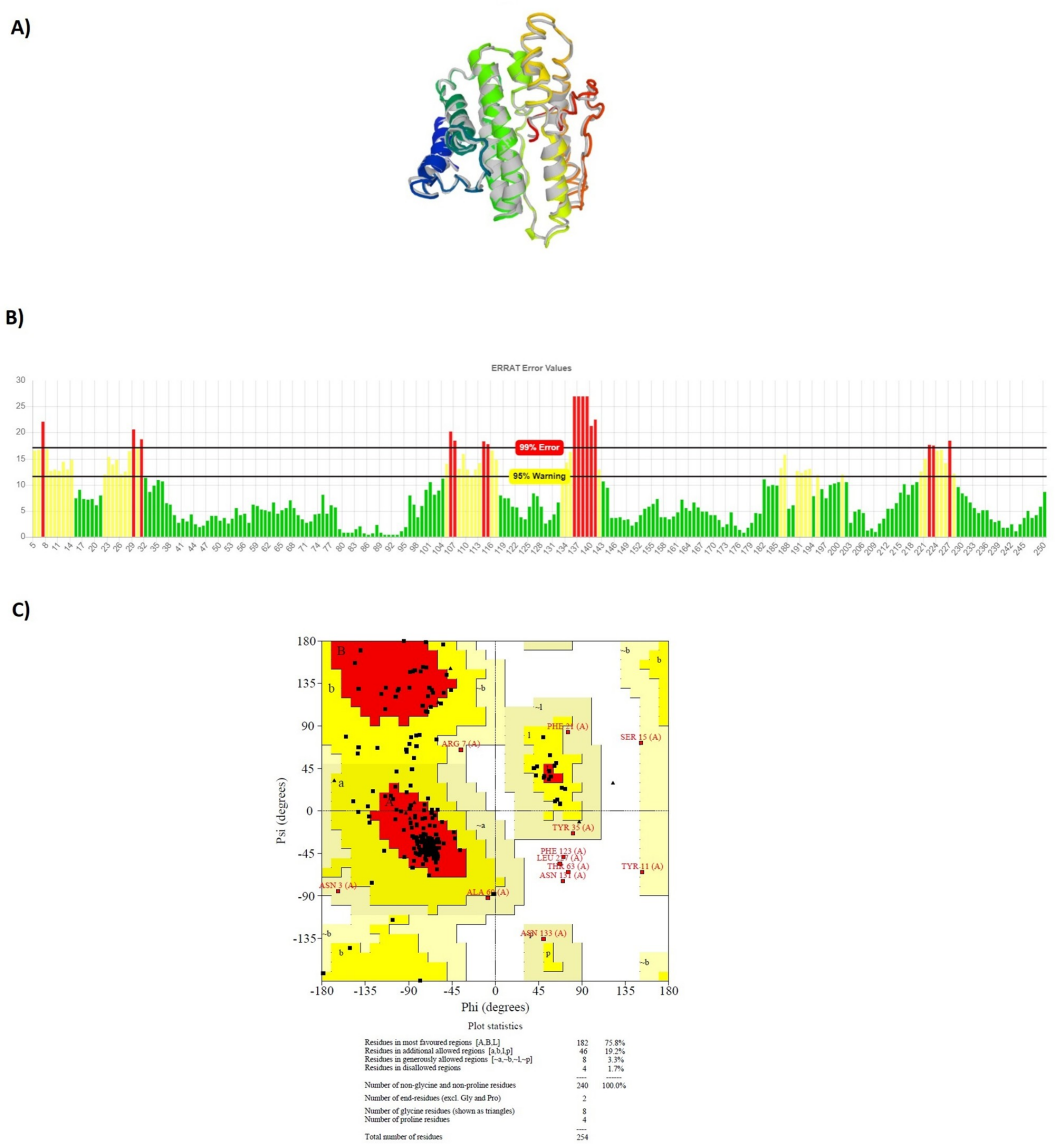
Refined characteristics of CTL multiepitope construct: a) Refined 3D prediction of CTL multiepitope construct, b) overall quality of refinement, c) Ramachandran plot.

**Fig 7:**
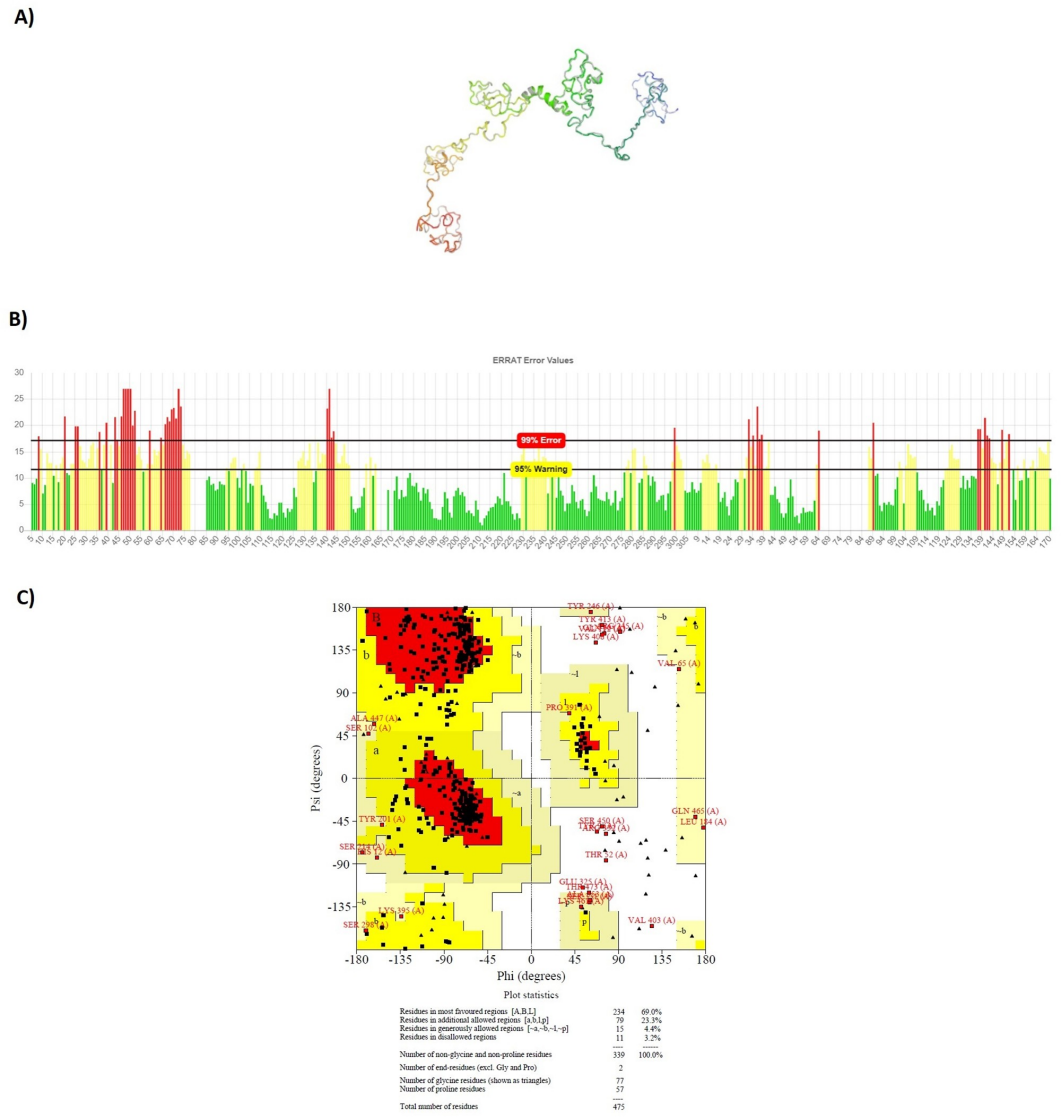
Refined characteristics of HTL multiepitope construct: a) Refined 3D prediction of HTL multiepitope construct, b) overall quality of refinement, c) Ramachandran plot.

After tertiary structure prediction, the top model of each construct was submitted separately to GalaxyRefine 2 server. GalaxyRefine server rebuilds side-chain, and performs side-chain repacking and structure relaxation by molecular dynamic simulation. After refinement process, refined models were submitted to SAVE5.05 server for validation. The data indicated that the quality of tertiary structure was improved after refinement process. Most of the residues were found in favored and allowed regions: 98.9% for LBL, 98.3% for CTL and 96.8% for HTL constructs. Figs 5–7 show refined characteristics including secondary structures, overall quality and Ramachandran plots.

### Prediction of discontinuous antibody epitopes

Linear antibody epitopes could be predicted through sequence-based algorithms. In contrast, prediction of discontinuous epitopes needs 3D structural information of the protein or polypeptide. Thus, the selected refined models were analyzed by Ellipro server to predict potential discontinuous B-cell epitopes. Ellipro servers identified 3 discontinuous B-cell epitopes for CTL, 4 for HTL and 3 for LBL. S1 Table indicates residues, number of residues and the 3D structure of putative B-cell epitopes in the designed constructs.

### Docking between vaccine constructs and Toll-Like Receptors (TLRs)

**Fig 8:**
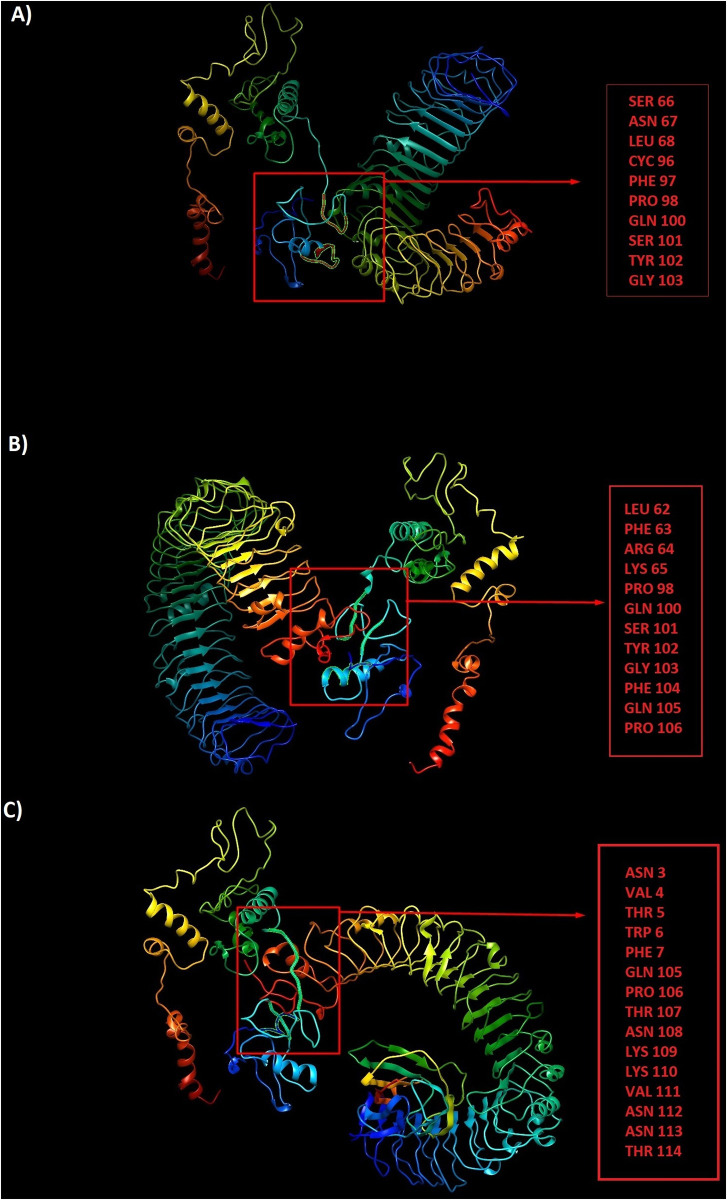
The peptide-protein docking between LBL construct and TLRs 2, 3 and 4: a) LBL-TLR2 complex with participated residues in interaction, b) LBL-TLR3 complex with participated residues in interaction, c) LBL-TLR4 complex with participated residues in interaction.

**Fig 9:**
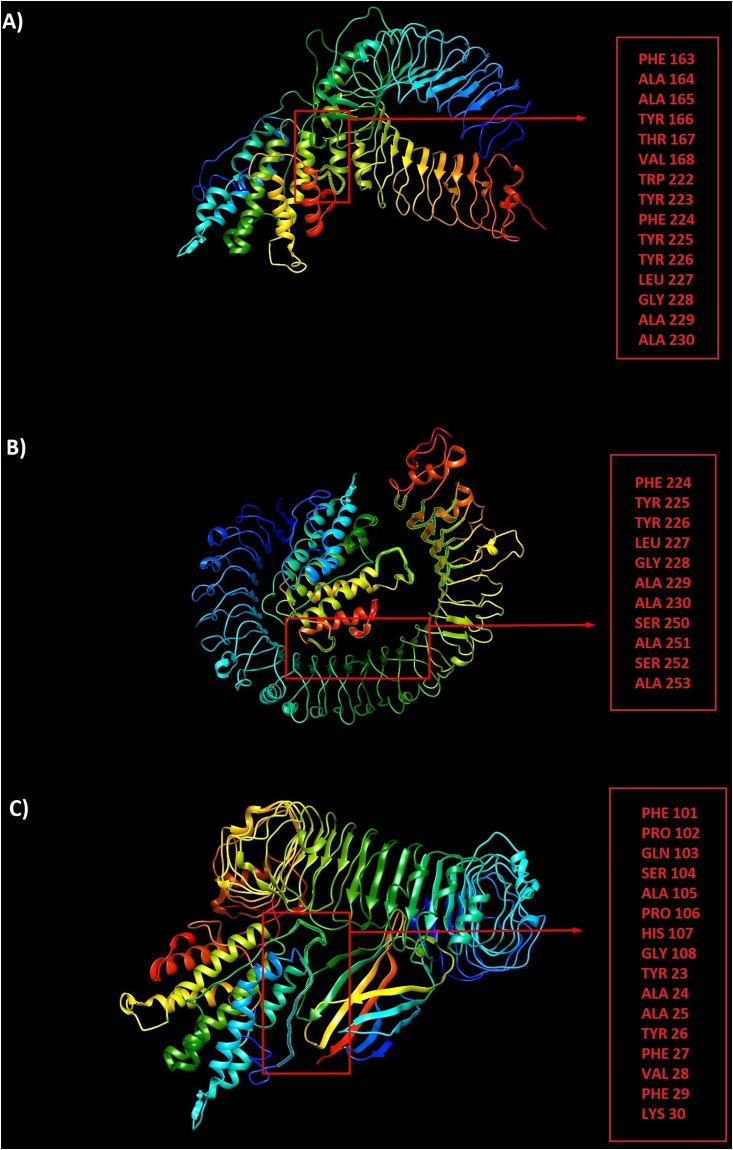
The peptide-protein docking between CTL construct and TLRs 2, 3 and 4: a) CTL-TLR2 complex with participated residues in interaction, b) CTL-TLR3 complex with participated residues in interaction, c) CTL-TLR4 complex with participated residues in interaction.

**Fig 10:**
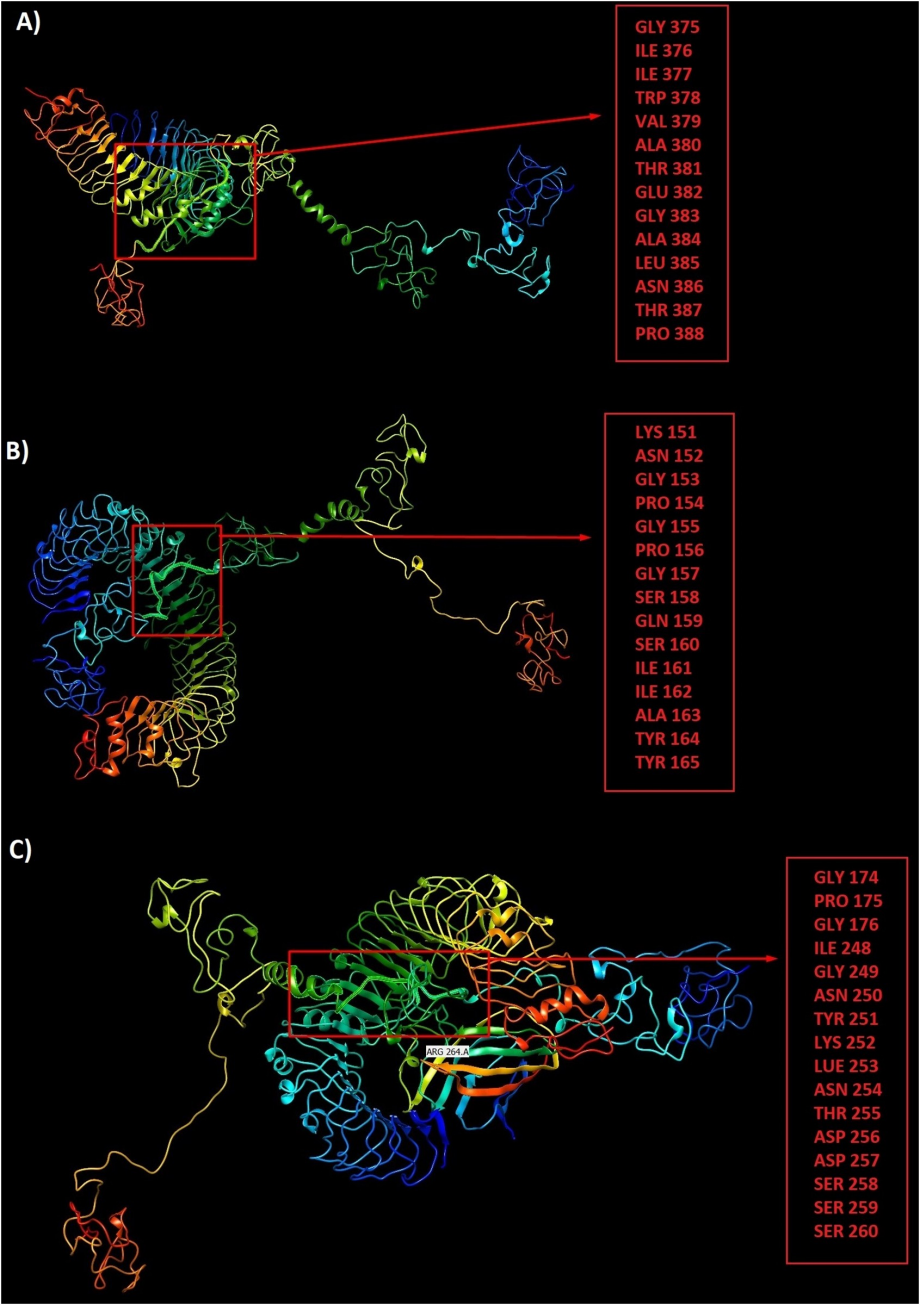
The peptide-protein docking between HTL construct and TLRs 2, 3 and 4: a) HTL-TLR2 complex with participated residues in interaction, b) HTL-TLR3 complex with participated residues in interaction, c) HTL-TLR4 complex with participated residues in interaction.

The peptide-protein docking between three vaccine constructs and TLRs 2, 3 and 4 were performed by ClusPro server. The lowest energy level was estimated for LBL-TLR2 complex-812.3, for LBL-TLR3 complex -894.8, for LBL-TLR4 complex -875.3, for CTL-TLR2 complex -1087.8, for CTL-TLR3 complex -1385.3, for CTL-TLR4 complex -1180.8, for HTL-TLR2 complex -1143.6, for HTL-TLR3 complex -1296.6 and for HTL-TLR4 complex -1119.6. Strong interactions between the designed constructs and TLRs 2, 3 and 4 supports the hypothesis of SARS-CoV-2 susceptibility to TLRs 2, 3 and 4 like other Coronaviridae families (Figs 8–10). All three constructs showed better interactions with TLR 3.

## Discussion

The SARS-CoV-2 has become a major global public health issue and scientists are struggling to find the best way to treat the disease and develop a vaccine against the virus. Numerous immune-bioinformatics methods have been developed in vaccine researches which can potentially save time and resources. These tools could help us to identify antigenic domains to design a multi-epitope vaccine. Since now we know enough information about SARS-CoV-2’s genomics and proteomics, we can design peptide vaccines based on a neutralizing epitope. These immunoinformatics methods have made a significant impact on the immunology researches and we can see many examples of *in silico* design of epitope-based vaccines against many viruses including human immunodeficiency virus (HIV) [16], human papillomavirus (HPV) [38, 39], SARS-CoV [40], rhinovirus [41].

SARS-CoV-2 is an RNA virus tending to mutate more frequently [42]. These mutations mostly occur at the surface of the protein like at S protein leaving the immune system in a blind spot. Being the main antigenic component, S protein of SARS-CoV-2 has been selected as an important target for vaccine development since it is a crucial factor modulating tropism and pathogenicity and has the ability to induce faster and longer-term immune response [43, 44]. Since the humoral response from memory B-cells can easily be overcome by the emergence of antigens, it is important to design constructs based on cell-mediated immunity leading to lifelong immunity. Thus, our *in-silico* approaches were intended to design a universal SARS-CoV-2 vaccine for induction of B- and T-cell immunity with efficient reactions to the virus and long-term immune responses based on the S protein of the virus and also two other structural proteins including N and M.

There are some efforts based on designing epitope-based vaccines against SARS-CoV-2 since the outbreak. In one study, Abdelmageed *et al*. suggested certain peptides in E protein of SARS-CoV-2 as promising epitope vaccine candidates against T-cell [45]. In another study, Singh *et al*. proposed one construct including E, S, N and M proteins as an epitope vaccine candidate [46]. On the other hand, Feng *et al*. suggested some putative B- and T-cell epitopes based on S, M and E proteins of SARS-CoV-2 [47]. Also, Enayatkhani *et al*. designed a multiepitope vaccine candidate based on N, M and open reading frame (ORF) 3a of SARS-CoV-2 [48], and Teimouri *et al*. tried to predict B- and T-cell epitopes of SARS-CoV-2 in comparison with SARS-CoV [49]. These papers contain some very worthwhile suggestions for ease of multi-epitope vaccine development and all of them demonstrate that a multi-epitope peptide vaccine targeting multiple antigens should be considered as an ideal approach for prevention and treatment of SARS-CoV-2.

According to the aforementioned finding, we tried to use computational and bioinformatics methods on the formulation of new SARS-CoV-2 vaccine against its structural proteins including S, N and M proteins in a more comprehensive way. In the beginning, the whole genome of SARS-CoV-2 was analyzed. Then, three major structural proteins including S, N and M were chosen for further analyses. We identified epitopes corresponding to B-cells and T-cells to design constructs being able to elicit both humoral and cellular immunity. We used BepiPred tool to predict putative B-cell epitopes and chose 8 putative epitopes of S and N protein being able to induce antibodies like IgG. In contrast, M protein of the virus could not induce any class of antibodies.

As CD8^+^ and CD4^+^ T-cells play a major role in antiviral immunity, we tried to evaluate the binding affinity to MHC class I and II molecules. Choosing S, N and M proteins of the virus as the antigenic site, we used NetMHCpan and NetMHCIIpan prediction tools to identify the most immunodominant regions. From all peptides predicted, we chose 20 putative epitopes for MHC class I and 20 putative epitopes for MHC class II. Since S protein is currently the most promising antigen formulation, we put the focus on the S protein epitopes and chose 10 epitopes of S protein and 10 epitopes of other proteins for each MHC class I and II. The IFN-γ and IL-10 cytokines were also measured as candidates in MHC class II epitopes as they promote the development of T-helper cells being required for B-cell, macrophage and cytotoxic T-cell activation. Among the CTL predicted epitopes, S^257-265^, S^603-611^ and S^360-368^ and among HTL predicted epitopes, N^167-181^, S^313-330^ and S^1110-1126^ had better MHC binding rank.

To predict antigen processing through the MHC class I antigen presentation pathway, we used NetCTL1.2 server. All of the predicted epitopes had upper cut off identification scores (> 0.75) showing a high quality of proteasomal cleavage and Tap transport efficiency. We also measured the epitopes for conservancy analysis. All predicted epitopes were 100% conserved within four different clades of SARS-CoV-2. In general, the selected epitopes had the potency to produce an immune response against S, V, G and O clades of SARS-CoV-2.

Population coverage is another important factor in vaccine design. We measured population coverage rate for CTL and HTL epitopes in 16 specified geographical regions. For CTL epitopes, and helper T-cell epitopes, the highest population coverage of the world’s population was calculated for S^27-37^ with 86.27%, and for S^196-231^, S^303-323^, S^313-330^, S^1009-1030^ and N^328-349^ with 90.33%, respectively. Overall, these results suggest a specific binding of CTL epitopes and HTL epitopes to the prevalent HLA molecules in the targeted populations. Another prominent obstacle in vaccine development is the probability of allergenicity since many vaccines stimulate the immune system into an allergenic reaction. In this study, we used PA^3^P to predict potential allergenicity and all of the epitopes were analyzed as non-allergen.

For CTL epitopes, N^103-113^, S^868-876^, M^36-46^, S^1094-1102^, M^102-111^, S^1051-1061^, S^360-368^, S^191-199^ and S^686-696^ had the highest average of interaction similarity score, respectively and For HTL epitopes, M^107-128^, N^48-63^, S^689-704^, S^1057-1074^, S^196-231^, N^328-349^, S^32-53^, N^126-143^, M^163-181^ and S^114-130^ had the highest average of interaction similarity score, respectively. Overall, CTL epitopes showed better quality of docking in comparison with HTL epitopes. Finally, the vaccine construction was completed after joining the LBL, CTL and HTL epitopes with KK, AAY and GPGPG linkers, respectively.

The molecular weights of the constructed LBL, CTL and HTL epitopes were obtained as 36.5, 28.6 and 49.5 kDa, respectively which were low molecular weights for a multiepitope vaccine. All constructs were soluble and stable indicating that the designed constructs had high solubility and stability for the initiation of an immunogenic reaction.

In the case of 3D modeling, we used I-TASSER server to predict the tertiary protein structure. The accuracy of the selected models was evaluated by C-score. The C-scores of the models for LBL, CTL and HTL polypeptide constructs were -2.39, -4.42 and -0.63, respectively. The higher value of the C-score is the better quality of prediction. Thus, HTL with the C-score of -0.63 showed higher accuracy of the predicted epitopes. Also, the quality of the predicted constructs was improved by refinement which leads to a higher quality of final models. Over the 96.8% of the residues were found in favored and allowed regions. At last, we used Ellipro server to predict potential discontinuous B-cell epitopes. Ellipro servers identified 3 discontinuous B-cell epitopes for CTL with 143 residues, 4 for HTL with 225 residues and 3 for LBL with 72 residues indicating the ability of the designed constructs for robust induction of humoral response. Also, peptide-protein docking between three vaccine constructs and TLRs 2, 3 and 4 were performed by ClusPro server, and all data showed strong interactions between the designed constructs and TLRs 2, 3 and 4 supporting the hypothesis of SARS-CoV-2 susceptibility to TLRs 2, 3 and 4 like other Coronaviridae family. All three constructs showed better interactions with TLR 3.

Overall, we tried to consider three major structural proteins including S, N and M proteins of the virus and design three different constructs including LBL, CTL and HTL constructs to elicit more robust humoral and cellular immunity. Comparing our study with other studies in the field of multi-epitope vaccine design for SARS-CoV-2, all LBL epitopes obtained in Table 1 were reported in Bhattacharya *et al*. paper using the same server of Bepipred [50]. However, we chose the ones being able to induce different classes of antibodies including IgG and IgA. Among CTL epitopes obtained in Table 2, S^686-696^ (VASQSIIAYTM), S^1051-1061^ (FPQSAPHGVVF), N^103-113^ (LSPRWYFYYLG), N^304-313^ (AQFAPSASAF) and all of M epitope of CTL including M^36-46^ (FAYANRNRFLY), M^168-180^ (TVATSRTLSYY), M^195-204^ (YSRYRIGNYK), M^90-99^ (MWLSYFIASF) and M^102-111^ (FARTRSMWSF) have not been reported in any literature. Also, among HTL epitopes reported in Table 3, S^303-323^ (LKSFTVEKGIYQTSNFRVQPT), S^1009-1030^ (TQQLIRAAEIRASANLAATKMS), S^801-817^ (NFSQILPDPSKPSKRSF), N^328-349^(GTWLTYTGAIKLDDKDPNFKDQ), N^126-143^ (NKDGIIWVATEGALNTPK), N^342-361^ (KDPNFKDQVILLNKHIDAYK), N^48-63^ (NTASWFTALTQHGKED), N^167-181^ (LPKGFYAEGSRGGSQ), N^405-419^ (KQLQQSMSSADSTQA), M^107-128^ (RSMWSFNPETNILLNVPLHGTI) and M^163-181^ (DLPKEITVATSRTLSYYKL) have not been reported in any literature. The rest of the epitopes mentioned in Tables 2 and 3 were found in agreement with Teimouri *et al*. [49] for MHC class I and Feng *et al*. [47] for MHC class II, respectively. Also, we found one putative CTL epitope, S^360-368^ (CVADYSVLY) related to receptor-binding domain (RBD) region for S protein which was referred to the fragment of 347 to 520 amino acids [51]. We also identified overall 10 discontinuous B-cell epitopes for three multi-epitope constructs. Meanwhile, we investigated the interaction of three designed constructs with TLRs 2, 3 and 4 based on the previous studies on other Coronaviridae family such as SARS-CoV and MERS-CoV [33–35]. All three constructs showed strong interactions with TLRs 2, 3 and 4 supporting the hypothesis of SARS-CoV-2 susceptibility to TLRs 2, 3 and 4 like other Coronaviridae families. Albeit, SARS-CoV-2 was identified for only 5 month, but researches have recently begun to design a multiepitope vaccine. Thus, the collected data and information are very limited and need to be accumulated to improve existing processes and the designed multi-epitope vaccine needs to be tested clinically to validate vaccine safety.

## Conclusion

In conclusion, we determined three vaccine constructs against three major structural proteins of SARS-CoV-2 designed based on robust vaccine design criteria including non-allergenicity, conservancy, affinity measurement to multiple alleles of MHC, worldwide population coverage, 3D prediction, refinement and validation, discontinuous B-cell epitope prediction, docking and effectiveness of molecular interaction with their respective HLA alleles and TLRs. These constructs require validation by *in vivo* and clinical experiments. Generally, with the help of *in silico* studies, experimental researches can march rapidly with higher probabilities of finding the desired solutions and controlling the current outbreak.

## Supporting information

S1 Table Discontinuous B-Cell epitope on HTL, CTL and LBL polyepitope constructs. (DOCX)
