# Supplementary material for: Design of novel multiepitope constructs-based peptide vaccine against the structural S, N and M proteins of human COVID-19 using immunoinformatics analysis
Source: PLoS One. 2020 Oct 15;15(10):e0240577. doi: 10.1371/journal.pone.0240577 (PMC7561160; doi:10.1371/journal.pone.0240577)
Supplement: S1 Table — (DOCX) [file pone.0240577.s001.docx]

| *S1 Table: Discontinuous B-Cell epitope on HTL, CTL and LBL polyepitope constructs* | | | |
| --- | --- | --- | --- |
| Constructs | **Residues** | **Number of residues** | **3D view** |
| CTL | A:R183, A:I186, A:G187, A:N188, A:Y189, A:K190, A:A191, A:A192, A:Y193, A:N194, A:T195, A:A196, A:S197, A:T200, A:W222, A:Y223, A:F224, A:Y225, A:Y226, A:L227, A:G228, A:A229, A:A230, A:Y231, A:T232, A:K233, A:A234, A:Y235, A:N236, A:V237, A:T238, A:Q239, A:A242, A:A243, A:A245, A:Q246, A:F247, A:A248, A:P249, A:S250, A:A251, A:S252, A:A253, A:F254 | 44 | 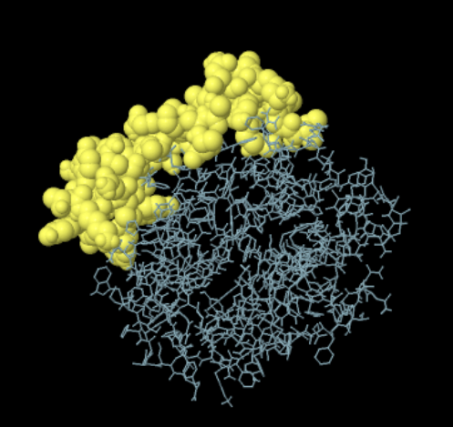 |
|  | A:Y129, A:A130, A:N131, A:R132, A:N133 | 5 | 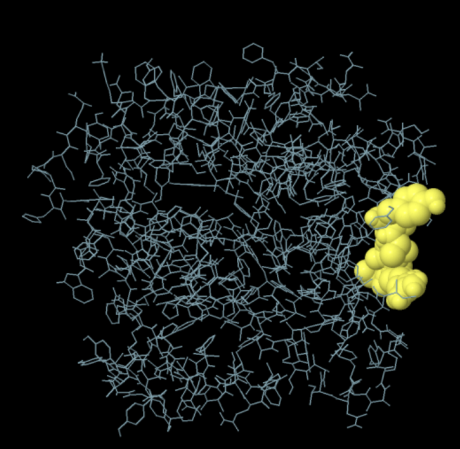 |
|  | A:R7, A:G8, A:V9, A:Y10, A:Y11, A:A12, A:A13, A:Y14, A:S15, A:A16, A:N17, A:N18, A:C19, A:T20, A:F21, A:E22, A:Y23, A:A24, A:A25, A:Y26, A:F27, A:V28, A:F29, A:K30, A:N31, A:I32, A:D33, A:G34, A:Y35, A:A36, A:A37, A:Y38, A:W39, A:T40, A:A41, A:G42, A:A43, A:A44, A:V57, A:L58, A:Y59, A:A60, A:A61, A:Y62, A:T63, A:S64, A:N65, A:Q66, A:V67, A:A68, A:V69, A:L70, A:Y71, A:A72, A:A73, A:Y74, A:V75, A:S77, A:Q78, A:S79, A:I80, A:I81, A:A98, A:A99, A:Y100, A:F101, A:P102, A:Q103, A:S104, A:A105, A:P106, A:H107, A:G108, A:V109, A:V110, A:R158, A:S159, A:M160, A:W161, A:S162, A:F163, A:A164, A:T210, A:Q211, A:H212, A:G213, A:K214, A:A215, A:A216, A:Y217, A:L218, A:S219, A:P220, A:R221 | 94 | 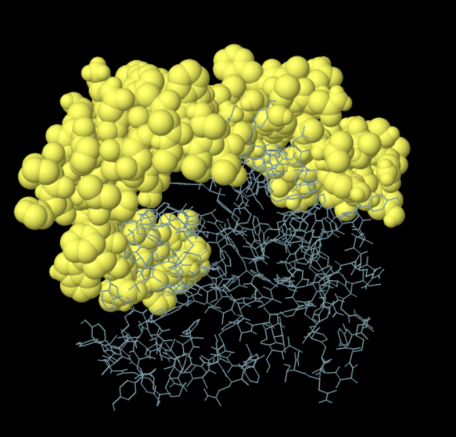 |
| HTL | A:N386, A:T387, A:P388, A:K389, A:G390, A:P391, A:G392, A:P393, A:G394, A:K395, A:D396, A:P397, A:N398, A:F399, A:K400, A:D401, A:Q402, A:V403, A:I404, A:L405, A:L406, A:N407, A:K408, A:H409, A:I410, A:D411, A:A412, A:Y413, A:K414, A:G415, A:P416, A:G417, A:P418, A:G419, A:N420, A:T421, A:A422, A:S423, A:W424, A:F425, A:T426, A:A427, A:L428, A:T429, A:Q430, A:H431, A:G432, A:K433, A:E434, A:D435, A:G436, A:P437, A:G438, A:P439, A:G440, A:L441, A:P442, A:K443, A:G444, A:F445, A:Y446, A:A447, A:E448, A:G449, A:S450, A:R451, A:G452, A:G453, A:S454, A:Q455, A:G456, A:P457, A:G458, A:P459, A:G460, A:K461, A:Q462, A:L463, A:Q464, A:Q465, A:S466, A:M467, A:S468, A:S469, A:A470, A:D471, A:S472, A:T473, A:Q474, A:A475 | 90 | 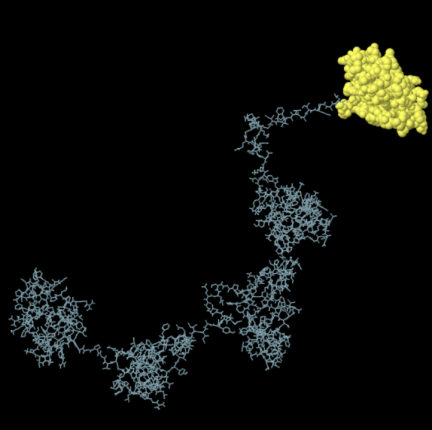 |
| Table continued | | | |
| HTL | A:N1, A:I2, A:D3, A:G4, A:Y5, A:F6, A:K7, A:I8, A:Y9, A:S10, A:K11, A:H12, A:T13, A:P14, A:I15, A:N16, A:L17, A:V18, A:R19, A:D20, A:L21, A:P22, A:Q23, A:G24, A:F25, A:S26, A:G27, A:P28, A:G29, A:P30, A:G31, A:L32, A:K33, A:S34, A:F35, A:T36, A:V37, A:E38, A:K39, A:G40, A:I41, A:Y42, A:Q43, A:T44, A:S45, A:N46, A:F47, A:R48, A:V49, A:Q50, A:P51, A:T52, A:G53, A:P54, A:G55, A:P56, A:G57, A:Y58, A:Q59, A:T60, A:S61, A:N62, A:F63, A:V65, A:Q66, A:P67, A:T68, A:E69, A:S70, A:I71, A:V72 | 76 | 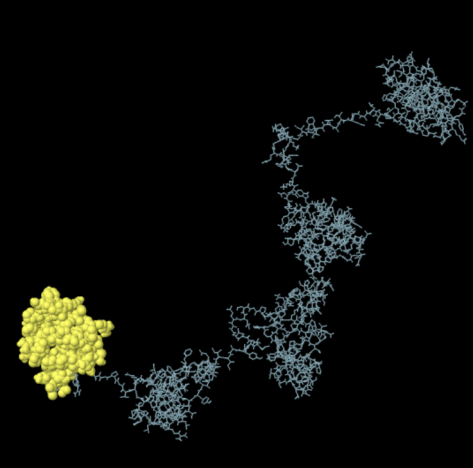 |
|  | A:P301, A:E302, A:T303, A:N304, A:I305, A:L306, A:L307, A:N308, A:V309, A:P310, A:L311, A:H312, A:G313, A:T314, A:I315, A:G316, A:P317, A:G318, A:P319, A:G320, A:D321, A:L322, A:P323, A:K324, A:E325, A:I326, A:T327, A:V328, A:A329, A:T330, A:S331, A:R332, A:T333, A:L334, A:S335, A:Y336, A:Y337, A:K338 | 38 | 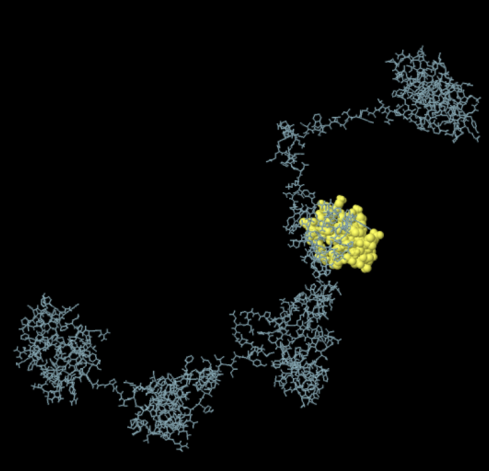 |
|  | A:Y114, A:P115, A:D116, A:K117, A:V118, A:F119, A:S121, A:S122, A:V123, A:L124, A:S126, A:T127, A:Q128, A:D129, A:G130, A:P131, A:G132, A:P133, A:G134, A:P135, A:H136 | 21 | 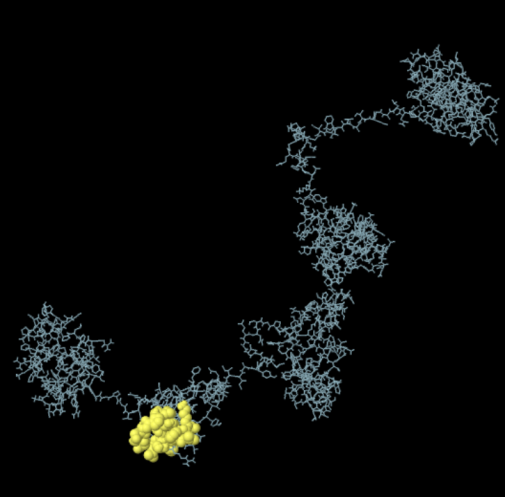 |
| LBL | A:A316, A:D317, A:E318, A:T319, A:Q320, A:A321, A:L322, A:P323, A:Q324, A:R325, A:Q326, A:K327 | 12 | 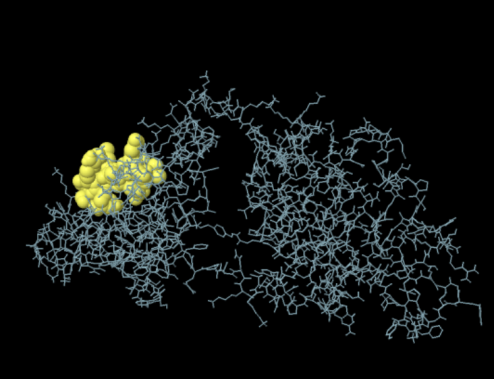 |
| Table continued | | | |
| LBL | A:K26, A:S27, A:F28, A:T29, A:V30, A:E31, A:K32, A:G33, A:I34, A:Y35, A:Q36, A:T37, A:S38, A:N39, A:N47, A:D49, A:S50, A:K51, A:V52, A:G53, A:G54, A:Y56, A:N57, A:Y58, A:Y60, A:R61, A:L62, A:F63, A:R64, A:K65, A:S66, A:N67, A:L68, A:K69, A:P70, A:F71, A:E72, A:S76, A:E78, A:Q81, A:P86, A:C87, A:N88, A:G89, A:V90, A:E91, A:G92, A:F93, A:N94, A:C95, A:Y96, A:F97, A:P98, A:L99, A:Q100, A:S101, A:Y102, A:G103 | 58 | 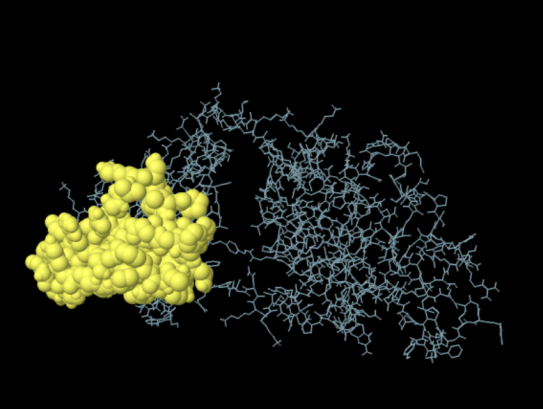 |
|  | A:P172, A:S173, A:D174, A:S175, A:T176, A:G177, A:S178, A:N179, A:Q180, A:N181, A:G182, A:E183, A:R184, A:S185 | 14 | 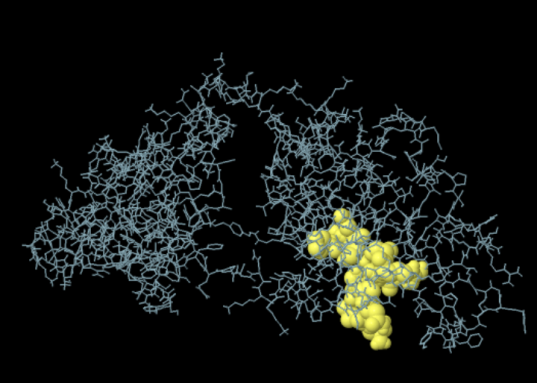 |
